# Room temperature three-photon pumped CH_3_NH_3_PbBr_3_ perovskite microlasers

**DOI:** 10.1038/srep45391

**Published:** 2017-03-28

**Authors:** Yisheng Gao, Shuai Wang, Can Huang, Ningbo Yi, Kaiyang Wang, Shumin Xiao, Qinghai Song

**Affiliations:** 1 State Key Laboratory on Tunable laser Technology, Ministry of Industry and Information Technology Key Lab of Micro-Nano Optoelectronic Information System, Shenzhen Graduate School, Harbin Institute of Technology, Shenzhen, 518055, China; 2 Collaborative Innovation Center of Extreme Optics, Shanxi University, Taiyuan, 030006, China

## Abstract

Hybrid lead halide perovskites have made great strides in next-generation light-harvesting and light emitting devices. Recently, they have also shown great potentials in nonlinear optical materials. Two-photon absorption and two-photon light emission have been thoroughly studied in past two years. However, the three-photon processes are rarely explored, especially for the laser emissions. Here we synthesized high quality CH_3_NH_3_PbBr_3_ perovskite microstructures with solution processed precipitation method and studied their optical properties. When the microstructures are pumped with intense 1240 nm lasers, we have observed clear optical limit effect and the band-to-band photoluminescence at 540 nm. By increasing the pumping density, whispering-gallery-mode based microlasers have been achieved from CH_3_NH_3_PbBr_3_ perovskite microplate and microrod for the first time. This work demonstrates the potentials of hybrid lead halide perovskites in nonlinear photonic devices.

Hybrid lead halide perovskites have been intensively studied due to their potential applications in photovoltaic devices^1^,^2^,^3^. In just four years, the light conversion coefficient has been dramatically improved from 3.5%^4^ to more than 22.1%^5^, which is comparable to or even larger than most of the thin film solar cells. In principle, the ferroelectric nature of hybrid lead halide perovskite has been considered to play an essential role in photovoltaic performances^6^,^7^. The ferroelectric domains help the separation of photo-excited electron and hole pairs, and the reduction of recombination through segregation of charge carriers^6^. The ferroelectric character can also induce quantum coherent wave propagation of exciton, which leads to more efficient charge extraction^7^. Interestingly, such kind of ferroelectric properties of hybrid lead halide perovskites also make them to be comparable to the other ferroelectric materials such as lithium niobate^8^,^9^,^10^,^11^,^12^. Consequently, the nonlinear optical properties can be expected in the hybrid lead halide perovskites. Within past two years, a number of nonlinear effects have been experimentally demonstrated, e.g. two-photon absorption^8^,^9^,^10^, two-photon pumped photoluminescence (PL)^8^, two-photon pumped amplified spontaneous emission (ASE)^13^, and two-photon pumped lasers^14^. Very recently, three-photon pumped PL and amplifications have also been reported^15^. However, compared with the two-photon process, the three-photon absorption and three-photon emission are higher order nonlinear process and have much smaller coefficients/cross sections. Till now, only the three-photon ASE has been observed in hybrid lead halide perovskite bulk^15^. Three-photon excited lasers are still absent, especially in the hybrid lead halide microstructures. Therefore, considering the progresses in water-resistant perovskite^16^,^17^,^18^, it is also very essential and highly desirable to develop a three-photon pumped hybrid lead halide perovskite microlasers at room-temperature. Here we have synthesized single-crystalline CH_3_NH_3_PbBr_3_ microstructures and demonstrated the three-photon excited lasing actions within them for the first time.

## Results and Discussion

**Figure 1:**
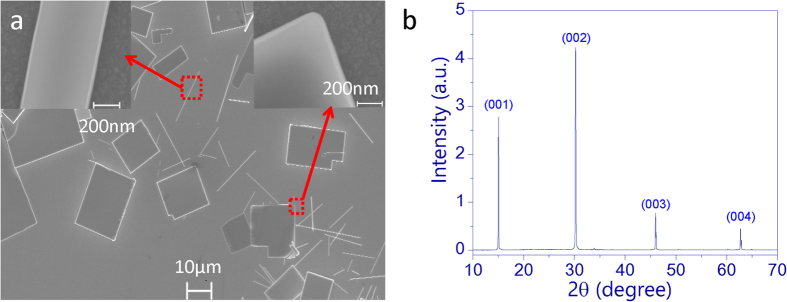
(**a**) Top-view SEM image of the synthesized microstructures. The insets show the high resolution SEM images of microplate and microrod. (**b**) XRD spectrum of CH_3_NH_3_PbBr_3_ microstructures.

Figure 1(a) shows the top-view scanning electron microscope (SEM) image of the synthesized microstructures. We can see that the synthesized microstructures are dominated by the microplates and microrods. The lengths of these microstructures are tens of microns. The widths of the microrods vary between a few to several microns. The thicknesses of two types of microstructures are quite different. The thicknesses of microplates are usually around several hundred nanometers to a few microns (see examples in Figure S4 in supplemental information). Figure 1(b) shows the recorded X-ray diffraction (XRD) spectrum of the synthesized microstructures. Sharp peaks can be clearly seen at around 15° and 30°, matching the cubic phase and indicating the single crystal very well. The insets in Fig. 1(a) are the high resolution top-view SEM images of a microplate and a microrod. We can see that the boundaries of perovskite microplate and microrod are extremely smooth and uniform. It is hard to see clear flaws and fluctuations even though the scar bar is already as small as 200 nm. Thus we know that the scattering loss at the side-facets are negligibly small and lasing actions are possibly formed within these high quality perovskite microstructures^19^.

**Figure 2:**
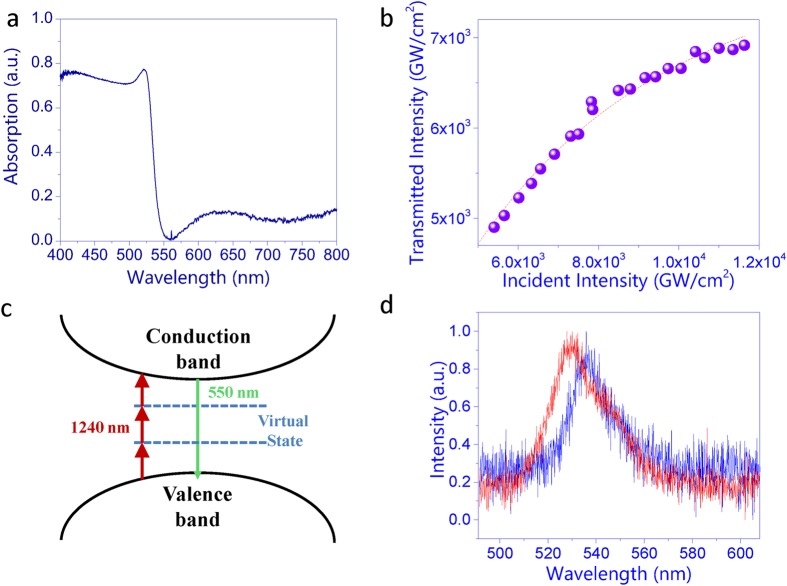
(**a**) Linear absorption of the CH_3_NH_3_PbBr_3_ microstructures. (**b**) The transmission of an ultrashort pulse at 1240 nm as a function of incident power. The dashed line represent the fitted curve following equation (2). (**c**) The schematic pictures of the three-photon absorption. (**d**) The PL spectra under one-photon and three-photon excitations.

Then the optical properties of synthesized microstructures were studied. Figure 2(a) shows the regular absorption spectrum that was recorded by measuring the linear transmission and refection spectra with a continue-wave white light source (see Figure S1 in supplemental information). We can see that the absorption drastically increases when the wavelength is below 550 nm (2.25 eV). For the wavelength range above 550 nm, the linearly material absorption is negligibly small. All these experimental observations are consistent with the previous reports very well^13^,^14^,^20^. The situation changed dramatically when the sample was illuminated with ultrashort laser pulses (100 fs, 1 KHz, see Figure S2 in supplemental information). One example is illustrated in Fig. 2(b), where the pumping wavelength is 1240 nm (1 eV). The transmitted energy increased linearly at low pumping density. With the increase of pumping power, the transmitted energy gradually deviated from the linear model (dashed line in Fig. 2(b)) and an obvious transmission limit can be observed. All these experimental results clearly indicate the occurrence of nonlinear absorption. Since the photon energy of pumping laser is smaller than half of the bandgap (2.25 eV) and larger than 1/3 of the bandgap, here the nonlinear absorption is induced by the three-photon process (see the corresponding schematic picture in Fig. 2(c)).

According to the basic theoretical consideration of three-photon, the intensity change of an excitation beam along the propagation direction (x direction) can be expressed as^21^


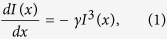


where γ is the three-photon-absorption coefficient of CH_3_NH_3_PbBr_3_ microplate. The solution of equation (1) can thus be obtained as


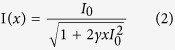


Therefore, the three-photon-absorption coefficient γ = 2.26 × 10^−5^*cm*^3^/*GW*^2^ have been obtained by fitting the data in Fig. 2(b).

The existence of three-photon absorption can also be confirmed from the emissions of the nanostructures. When the microstructures were excited with an ultrashort pulsed laser at 1240 nm, green light has been clearly observed (see the optical setup in Figure S3 supplemental information). Figure 2(d) shows the PL spectra under three-photon excitations. We can see that the central wavelength slightly shifts to longer wavelength about 10 nm and the full width at half maximum (FWHM) decreases from ~30 nm to ~20 nm. Similar to the two-photon process, both of the redshift and decrease in FWHM are also caused by the re-absorption of three-photon emission in hybrid halide perovskites^22^.

**Figure 3:**
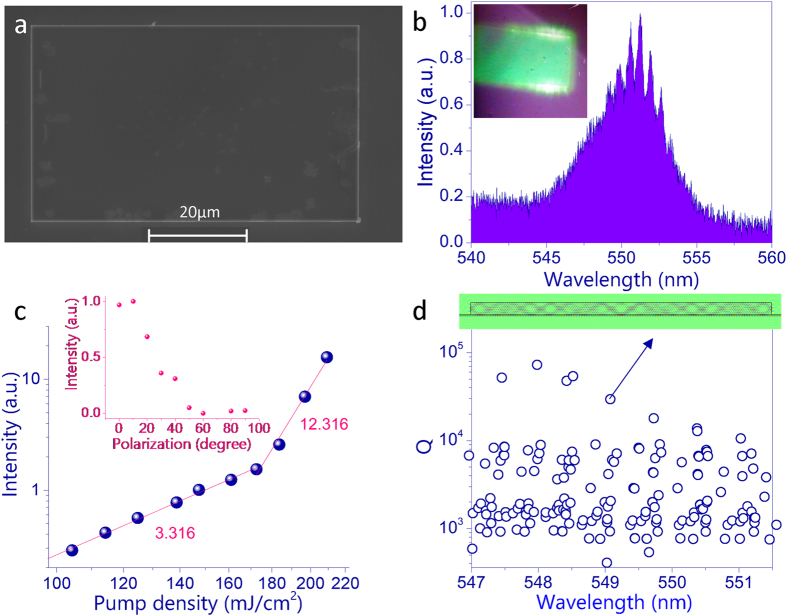
(**a**) Top view SEM image of a rectangle CH_3_NH_3_PbBr_3_ microplate. (**b**) The emission spectrum of the microplate with the pumping density at 197 mJ/cm^2^. The inset is the corresponding fluorescent microscope image. (**c**) The integrated output intensity as a function of pumping density. Inset shows the polarization of emitted laser in (**b**). (**d**) The numerical calculated cavity Q factors within the lasing wavelength range. The inset is the field pattern (Ez) of mode-1.

Based on the information on three-photon absorption and PL, the three-photon excited lasing action has been studied in one hybrid lead halide perovskite based microplate. The corresponding top-view SEM image is shown in Fig. 3(a). We can see that the microplate has a rectangle shape. The length and width of the rectangle are 65.4 microns and 39.2 microns, respectively. The thickness, which is measured by alfa stepper, is 1600 nm (see Figure S4 in supplemental information). When the pumping density was low, the emission spectrum was a broad spontaneous emission peak, which was similar to the one in Fig. 2(d). In the log-log plot, the integrated output intensity increases linearly with the pumping density. As shown in Fig. 3(c), the power slope of fitting line is 3.1, which is another direct evidence for the existence of three-photon absorption and PL^15^,^21^. Once the pumping power was above 170 mJ/cm^2^, the power slope drastically increases from around 3.3 to around 12.3. Meanwhile, sharp laser peaks emerged and quickly dominated the emission spectrum (see Fig. 3b). The emergence of sharp peaks and the changes in power slope clearly confirmed the three-photon pumped lasing action in CH_3_NH_3_PbBr_3_ microplate^23^,^24^,^25^. While the three-photon ASE has been reported in CH_3_NH_3_PbI_3_ bulk^15^, this is the first time that three-photon excited micro-lasers have been demonstrated, especially at the room temperature.

Figure 3(b) depicts the spectrum of three-photon laser emissions from the CH_3_NH_3_PbBr_3_ microplate. We can see a series of periodic peaks in the laser spectrum. The recorded mode spacing is 0.68 nm. The inset in Fig. 3(b) shows the corresponding fluorescent microscope image. With the onset of three-photon lasers, bright spots have also been seen at two end-facets of the short side. Based on the previous reports, it is easy to know that the three-photon lasers are generated along the short side. The polarization of emitted lasers were also recorded and plotted as inset in Fig. 3(c). The main polarization is along the long side of microplate. Thus we know that the transverse electric (TE, E is in-plane) polarized lasers have been formed.

As the conventional microlasers are one-to-one correspondence with the resonant modes, we thus numerically calculated the eigenmodes inside the microplate to understand the experimental observations. All the numerical calculations were performed with a finite element method based software package (Comsol Multiphysics 3.5a). Because the lasers were formed within the pumped strip along the short side, the system can be simplified into a two-dimensional rectangle in the transverse plane (see the details in Figure S5 in supplemental information)^26^,^27^. Figure 3(d) shows the numerically calculated quality (Q) factors of resonances inside the cavity. Here the cavity structures and refractive index were all taken from experimental data and material dispersions have been considered^28^. Due to the relative large refractive index of hybrid lead halide perovskite, the cavity can support a number of resonant modes. Around the lasing wavelength, a number of resonant modes marked 1–5 have relatively high Q factors and are easily excited in optical experiment. The mode spacing of modes 1–5 are around 0.65 nm, which is very close to the experimental result. Thus we know that the resonances 1–5 correspond to the lasing modes well. The inset in Fig. 3(d) shows the field pattern of resonance 2. Different from the conventional Fabry-Perot modes, we find that the light has an incident angle around 45 degree on two end-facets. Considering the large refractive index, the light is totally reflected and the resonances are whispering gallery like modes. For such kind of rectangle resonator, the main leakages are the boundary wave leakage and the pesudointegrable leakage^29^,^30^,^31^.

**Figure 4:**
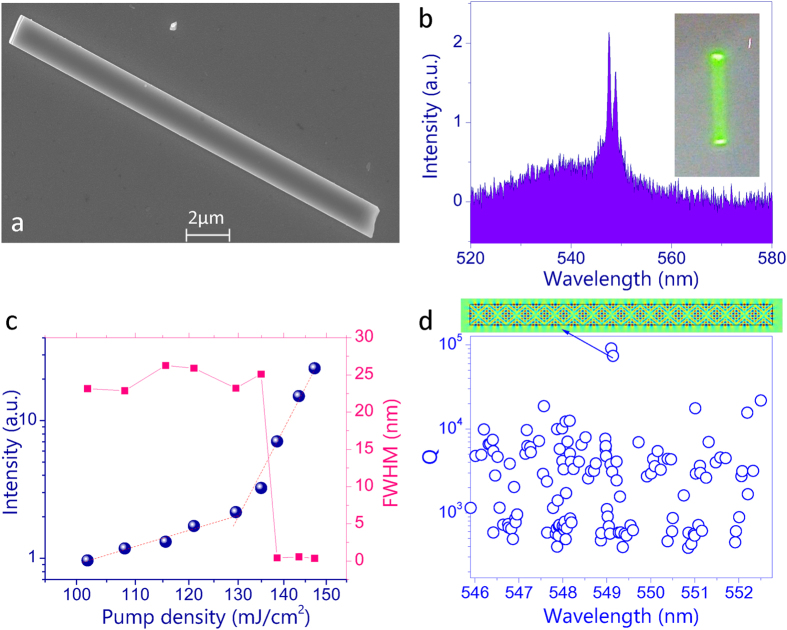
(**a**) Top view SEM image of a CH_3_NH_3_PbBr_3_ microrod. (**b**) The emission spectrum of transverse lasing mode in the microrod with the pumping density at 138.5 mJ/cm^2^. Inset shows the fluorescent microscope image. (**c**) The dependence of output intensity (dots) and the linewidth (squares) on the pumping density. (**d**) The numerical calculation results of resonant modes in microrod. Inset is the field pattern (Ez) of the highest Q mode.

In additional to the three-photon lasers in CH_3_NH_3_PbBr_3_ microplates, we have also studied three-photon lasing action in CH_3_NH_3_PbBr_3_ microrods. One example is summarized in Fig. 4. The top-view SEM image of the microrod is shown in Fig. 4(a). The length of microrod is 18.7 microns and its width is 1300 nm. The thickness of microrod is around 835 nm, which is shown in Figure S4 in supplemental information. Figure 4(b) shows the three-photon emission spectra under different pumping conditions. When the pumping power was low, a broad PL peak was seen at 547 nm (see Fig. 2(d) as an example) with full width at half maximum around 20 nm. With the increase of pumping power to above 130 mJ/cm^2^, periodic peaks appeared and quickly dominated the emission spectrum (see Fig. 4(b)). Figure 4(c) summarizes the output intensity as a function of pumping density. When the pumping power was low, the output intensity increased with a power slope ~3.3 in log-log plot, clearly demonstrating the three-photon PL process. When the pumping density was above 130 mJ/cm^2^, a dramatic change in power slope was observed. Meanwhile, the full width at half maximum of emission spectrum decreases from ~20 nm to ~0.3 nm, giving a Q factor around 1800. As mentioned above, the dramatic change in power slope and linewidth clearly demonstrated the onset of three-photon pumped microlaser.

The inset in Fig. 4(c) shows the fluorescent microscope image of the microrod with pumping density of 138.5 mJ/cm^2^. Similar to Fig. 3, bright spots can be observed at the end-facets of the CH_3_NH_3_PbBr_3_ microrod. This image indicated that the lasers were formed along the axial direction of the microrod. This is different from the transverse mode in Fig. 3. Another difference lies in the polarization. As shown in Figure S6 in the supplemental information, the polarization of three-photon microrod laser is following the axial direction, demonstrating the transverse magnetic (TM) polarization well.

Based on above information, we have also numerically simulated the resonances inside the system. All the results are shown in Fig. 4(d). While a large number of resonances have been formed around lasing wavelength range, there are several modes have much higher Q factors and have similar mode spacing with the experimental results. The inset in Fig. 4(d) shows the field pattern (Ez) of the highest Q resonant mode. We can see that this mode is also confined by total internal reflection at two end-facets. Compared with the modes in Fig. 3(d), here mode coupling happens^30^,^31^ and the field distributions are pushed away from the corners. Consequently, the boundary wave leakage is suppressed and the laser threshold in Fig. 4(c) was relatively lower than the one in Fig. 3(c).

## Conclusion

In summary, we have studied the three-photon process in single crystal CH_3_NH_3_PbBr_3_ microstructures. The measured three-photon-absorption coefficient was as large as. γ = 2.26 × 10^−5^*cm*^3^/*GW*^2^ Based on the large three-photon-absorption of hybrid lead halide perovskites, three-photon excited whispering-gallery-mode lasers have been experimentally observed in both of perovskite microplate and microrod for the first time. This research shows that the hybrid lead halide perovskite has very large fifth-order nonlinearity, which will be essential for practical applications such as optical switch. Compared with the two-photon process, three-photon absorption, PL, and lasers have much longer pumping laser wavelengths and the pumping lasers can propagate longer in highly scattering medium or cells. Therefore, considering the rapid developments in water resist lead halide perovskite, this research will also be important for developing active devices.

## Methods

### Fabrication

We synthesized the single crystalline CH_3_NH_3_PbBr_3_ microstructures with a solution based precipitation process^14^,^20^. Basically, CH_3_NH_3_Br (0.04 mmol) and PbBr_2_ (0.04 mmol) were dissolved in N, N-dimethyformamide (DMF) at a concentration of 0.02 M independently. Two solutions were mixed at room temperature (humidity 50%) with a 1:1 volume ratio. And a CH_3_NH_3_Br∙PbBr_2_ solution with a concentration at 0.01 M has been formed. The diluted solution was dip-casted onto an ITO-coated glass substrate which was fixed at a teflon stage. Then the teflon stage was sealed in a beaker with Dichloromethane (DCM = CH_2_Cl_2_). After 36 h, hybrid lead halide perovskites (CH_3_NH_3_PbBr_3_) microstructures were successfully synthesized on the substrate.

### Simulation and Measurement

The simulation of microplate and microrod were calculated by COMSOL MULTIPHYSICS and the experiment results were measured using an analog microscope system built by ourselves. The details of simulation and measurements are showed in support information.

## Additional Information

**How to cite this article**: Gao, Y. *et al*. Room temperature three-photon pumped CH_3_NH_3_PbBr_3_ perovskite microlasers. *Sci. Rep.* **7**, 45391; doi: 10.1038/srep45391 (2017).

**Publisher's note:** Springer Nature remains neutral with regard to jurisdictional claims in published maps and institutional affiliations.

## Supplementary Material

Supplementary Materials
