# Supplementary material for: Room temperature three-photon pumped CH3NH3PbBr3 perovskite microlasers
Source: Sci Rep. 2017 Mar 28;7:45391. doi: 10.1038/srep45391 (PMC5368977; doi:10.1038/srep45391)
Supplement: Supplementary Materials [file srep45391-s1.pdf]

Supplemental information: Room temperature three-photon pumped $CH_3NH_3PbBr_3$ perovskite microlasers

Yisheng Gao [1], Shuai Wang[2], Can Huang[2], Ningbo Yi[1], Kaiyang Wang[2], Shumin Xiao[1] and Qinghai Song[2 #]*

[1]Department of Material Science and Engineering, Harbin Institute of Technology, Shenzhen, 518055, China.

[2]National Key Laboratory on Tunable Laser Science and Technology, College of Science, Harbin Institute of Technology, Shenzhen, 518055, China.

* Email: shumin.xiao@hitsz.edu.cn

# Email: qinghai.song@hitsz.edu.cn

Figure S1: The schematic of measuring transmission of synthesized microstructures.

Figure S2: The schematic of measuring three-photon absorption.

Figure S3: The schematic of measuring three-photon laser.

Figure S4: (a) Thickness of the microplate measured by alfa stepper; (b) Section analysis of the microrod measured by AFM.

Figure S5: Schematic of numerical calculation.

Figure S6: The polarization of microrod.

Figure S7: The threshold curves of a single-photon laser and three-photon laser

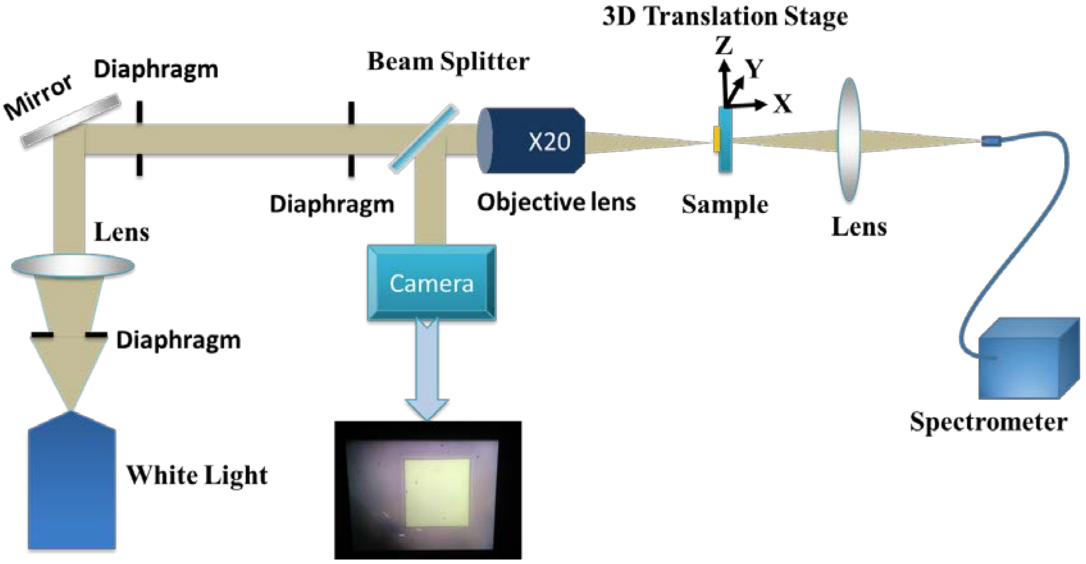

Figure S1: The schematic of measuring transmission of synthesized microstructures.

The white light was collimated by a conventional lens after passing a diaphragm. The collimated light passed a beam splitter and focused by a long working distance objective lens onto the top surface of perovskite microstructure, which was fixed on a three-dimensional translation stage. The reflected light was collected by the same objective lens and reflected to a CCD camera by the beam splitter. The relative position between the focus light spot and microstructure was controlled by the translation stage. The transmitted light was collected by another optical lens and coupled to a spectrometer via a multimode fiber. The transmittance was calculated with the equation $T = (I_{sam} - I_{dark})/(I_{sub} - I_{dark})$. Here I_{dark} was the intensity of background, I_{sub} intensity of spectrum after passing the substrate, and I_{sam} was the

intensity passing through the sample. Given the fact that reflection was small, we simply calcualted the absorption as $A = 1 - \mathrm{T}$.

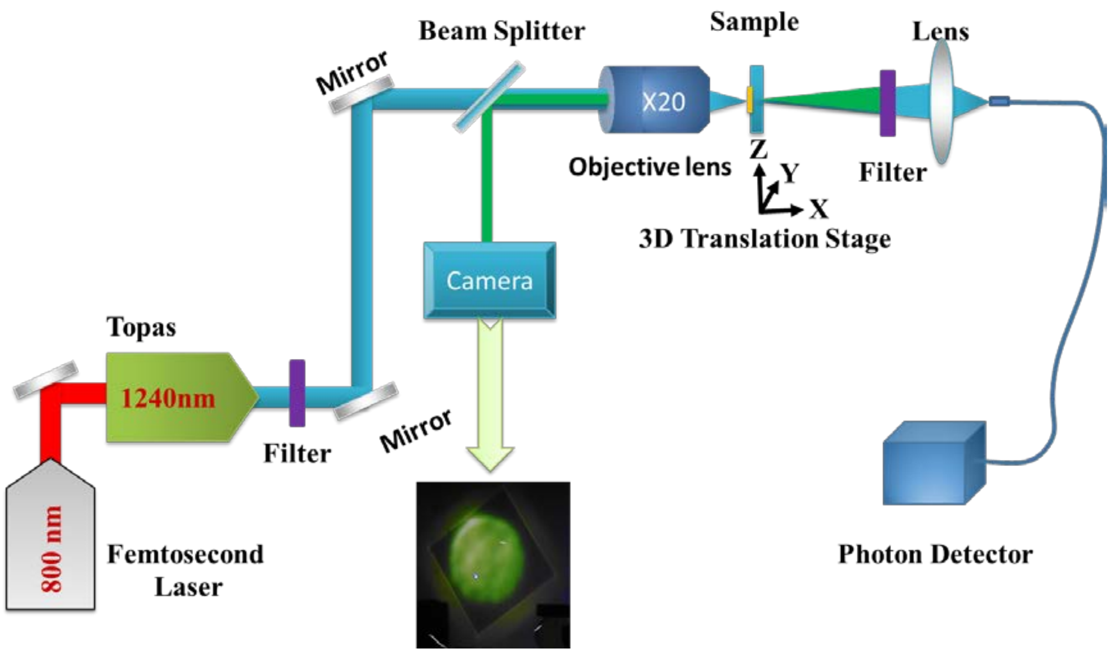

Figure S2: The schematic of measuring three-photon absorption.

As Figure S2 shows, the pump laser at 1240 nm from TOPAS empowered by ultrashort laser pulses (100 fs, 1 KHz) was focused on perovskite sample by a 20x objective lens with long working distance. The sample can be moved by a 3D translation stage. Behind the sample, the pump light was collected by a lens and detected by a photon detector. Two filters were used to purify pump light. At the same time a camera was utilized to observe the sample, just as the insets showed. To ensure the nonlinearity measurement, the pumping laser spot was adjusted to be smaller than the hybrid lead halide perovskite microplate.

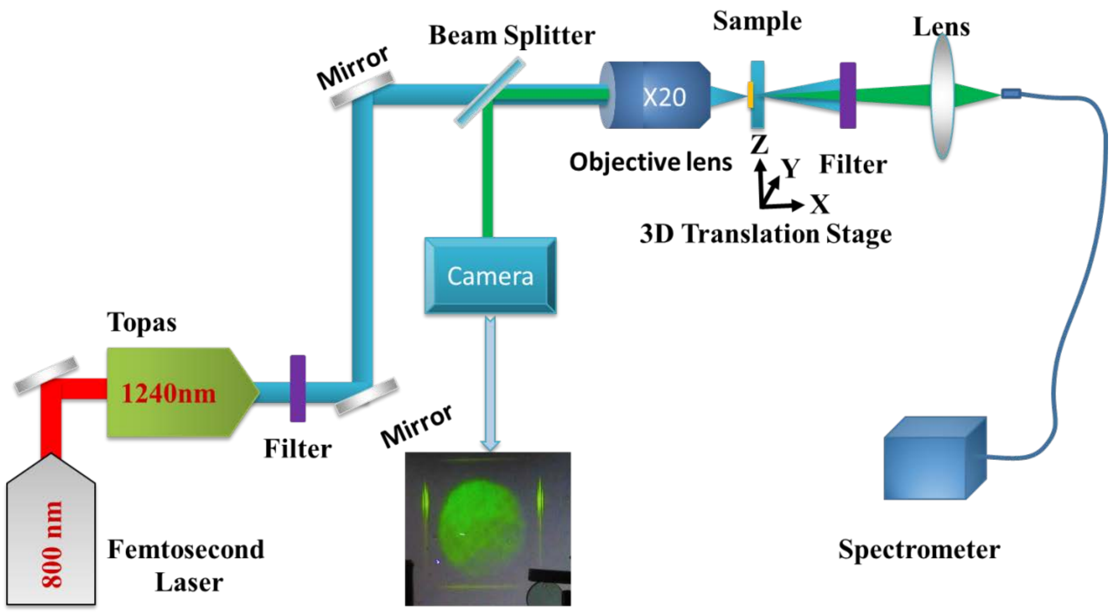

Figure S3: The schematic of measuring three-photon laser.

In the lasing experiment, the setup was pretty much similar to Figure S2. The differences were a shortpass filter was applied to block the pumping laser and the photodetector was replaced with a CCD (Princeton Instruments, PIXIS CCD) coupled spectrometer (Spectropro SP2700).

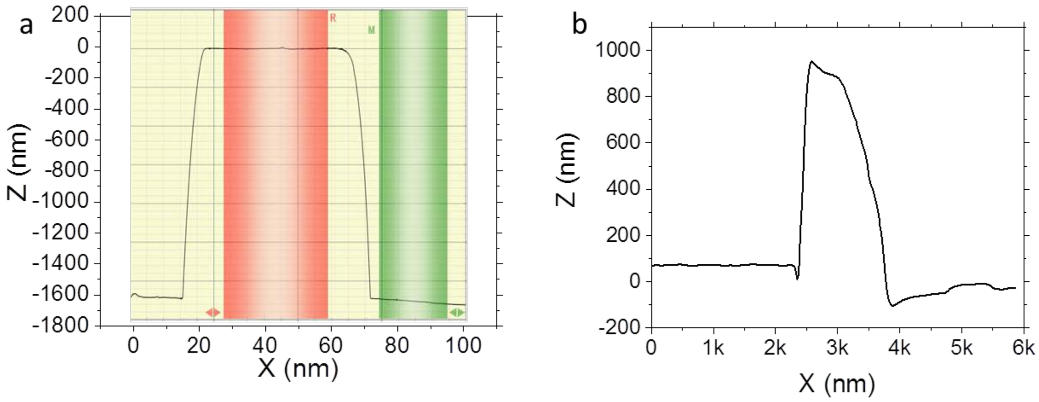

Figure S4: (a) Thickness of the microplate measured by alfa stepper; (b) Section analysis of the microrod measured by AFM.

In the main text, we have mentioned the thicknesses of the synthesized perovskite microstructure. Here we show two examples. As the left panel shown in Figure S4, the thickness of microplate is about 1600nm that was measured by alfa stepper. Due to the relatively small cross sections, the thickness of microrod was measured by atomic force microscope. As the right panel shown in Figure S4, the thickness is about 830nm according to section analysis.

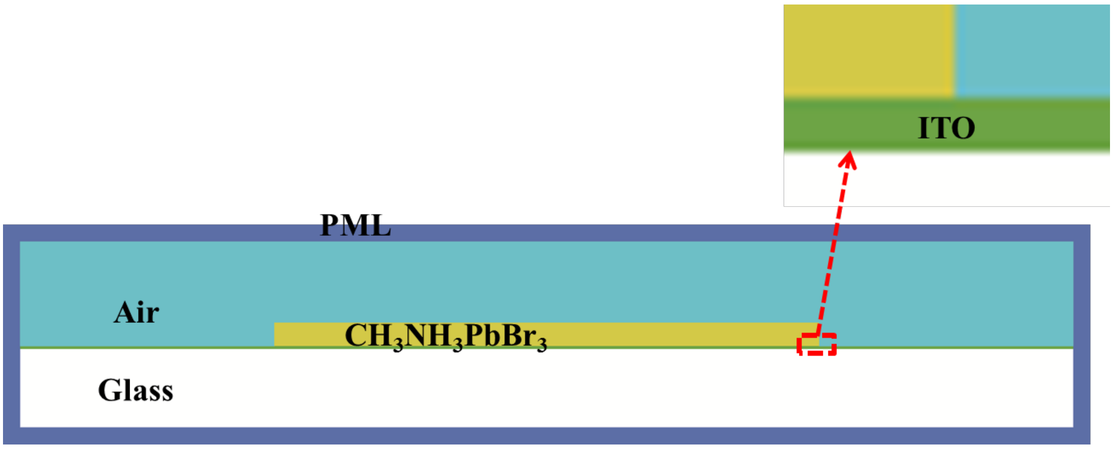

Figure S5: Schematic of numerical calculation.

In our numerical calculations, the three-dimensional microplate was simplified into a two dimensional object and simulated with a finite element method software package. The simplified structure was schematically illustrated in Figure S5. It is a perovskite structure (yellow region) that was placed on ITO-glass substrate (green and white areas). The reflective index of perovskite was taken from experimental results. The permittivity of ITO layer was described using the Drude model, and the reflective index of glass was fixed at 1.52. Thickness of perovskite structure was taken from the experimental results and the thickness of ITO was 180 nm. The perovskite was coated by an air layer (blue area, with reflective index

fixed at 1). During the numerical calculation, the structure was surrounded by perfect matching layer to absorb the outgoing waves. Complex numbered eigenfrequencies (w) have been obtained. The Q factors were calculated with $Q = \frac{Re(\omega)}{2|Im(\omega)|}$.

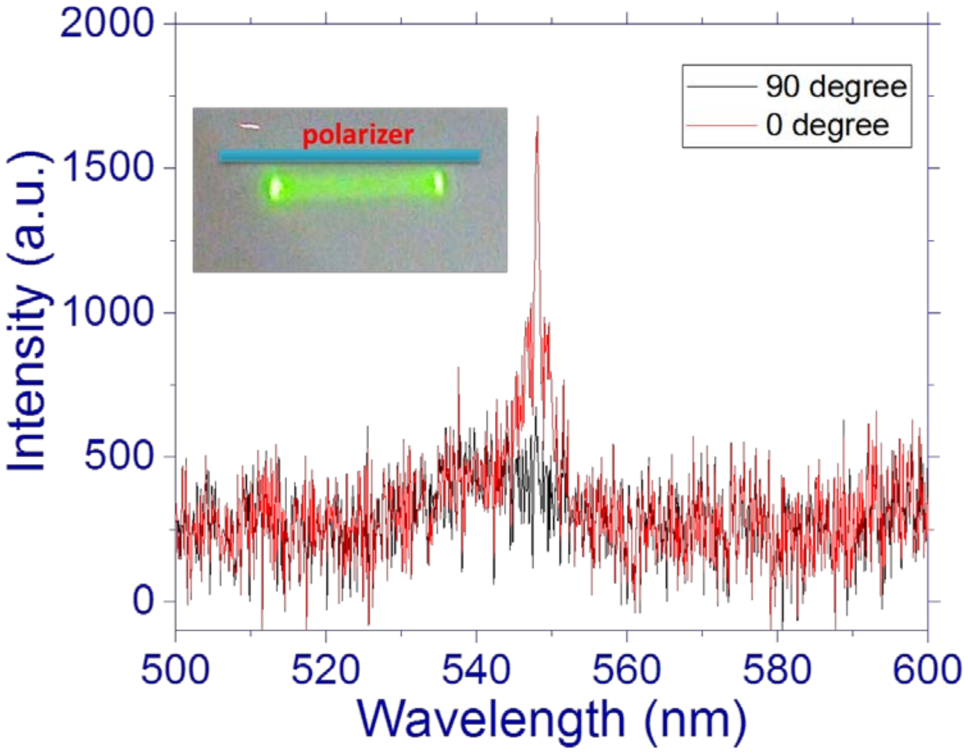

Figure S6: The polarization of microrod.

In this main text, we have mentioned the polarization of the three-photon microrod lasers. The experimental results are shown in Figure S6. The red line represents the polarization along the microrod, whereas the black line shows the spectrum with polarization perpendicular to the microrod. We can clearly see that the microrod lasers are primarily polarized along the axial direction, demonstrating the transverse magnetic (TM, E is perpendicular to the substrate) polarization well.

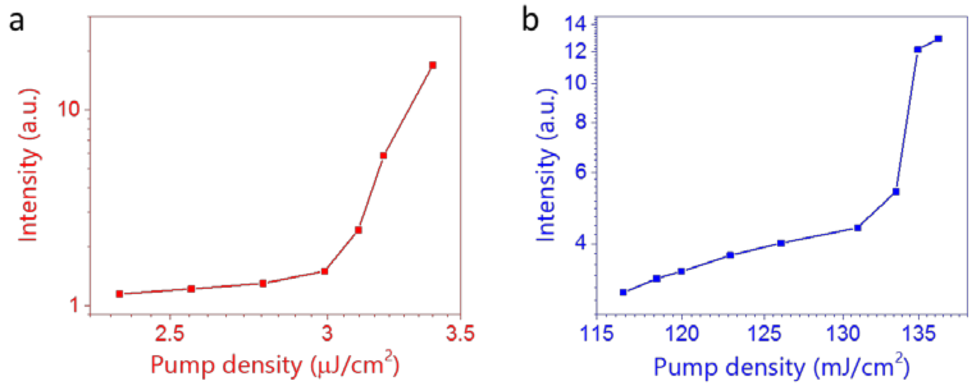

Figure S7 The relation between output intensity and pump density for a) single-photon excited at 400nm. b) three-photon laser excited at 1240nm

The single-photon and three-photon laser was compared on a same perovskite sample. Figure S7 shows the relations of output intensity and pump density under the pumping with 400nm and 1240nm lasers. It can be found that threshold of three-photon laser was much higher than that of single-photon laser.
